# Interplay between the static ordering and dynamical heterogeneities determining the dynamics of rotation and ordinary liquid phases in 1,6-anhydro-β-D-glucose

**DOI:** 10.1038/srep42103

**Published:** 2017-02-06

**Authors:** O. Madejczyk, K. Kaminski, E. Kaminska, K. Jurkiewicz, M. Tarnacka, A. Burian, M. Paluch

**Affiliations:** 1 Institute of Physics, University of Silesia, ul. Uniwersytecka 4, 40-007 Katowice, Poland; 2 Silesian Center for Education and Interdisciplinary Research, University of Silesia, ul. 75 Pulku Piechoty 1A, 41-500 Chorzow, Poland; 3 Department of Pharmacognosy and Phytochemistry, Medical University of Silesia in Katowice, School of Pharmacy with the Division of Laboratory Medicine in Sosnowiec, ul. Jagiellonska 4, 41-200 Sosnowiec, Poland

## Abstract

In this letter, we reported thorough the structural and molecular dynamics studies on 1,6-anhydro-β-D-glucose, the second compound reported so far that is capable to form rotator and supercooled liquid phases. In contrast to the data presented for ethanol, temperature dependences of structural dynamics in both phases are very comparable. On the other hand, X ray measurements revealed unusually long range ordering/correlations between molecules in the ODIC (*d* ≈ 95 Å) and supercooled phases (*d* ≈ 30–40 Å) of this carbohydrate. Our consideration clearly demonstrated that the interplay between length scales of static range ordering and dynamical heterogeneities as well as internal molecular arrangement seem to be the key to understanding the molecular dynamics of different materials characterized by varying degree of disorder in the vicinity of the glass transition temperature.

The molecular origin of the glass transition phenomenon seems to be one of the most challenging issue of condensed matter physics. The special effort is put to understand the microscopic nature of this peculiar phase transition as well as to explain enormous increase of the viscosity or relaxation times in the close vicinity of the glass transition temperature, *T*_g_, probed by different spectroscopies, including mechanical, dielectric or dynamic light scattering ones^1^,^2^,^3^,^4^. To get deeper insight into nature of this characteristic feature of supercooled liquids, systematic theoretical and experimental studies on various kinds of materials characterized by different degree of disorder and intermolecular interactions have been performed^5^,^6^,^7^. In this context, it is worthwhile to mention Orientationally Disordered Crystals (ODIC) that seem to be very promising materials, having centers of mass of molecules fixed like in the crystalline lattice. Simultaneously, they are characterized by the orientational disorder. Interestingly, the current studies show that in many aspects plastic crystals behave in the same way as it was reported for the ordinary glass formers^8^,^9^,^10^. Just to mention that both kinds of materials reveal clear deviation from the Arrhenius-like behavior of the temperature dependence of structural relaxation times. Although, a degree of deviation, as quantified by the fragility index, (*m)*, is much smaller in the case of plastic crystals. The best illustration of this relationship is ethanol, the only liquid that can form PC and SL phases at the same thermodynamic conditions. Over the years, this alcohol became a canonical reference material for the discussion of the data measured for other plastic crystals. Moreover, taking into account the behavior of ethanol, low fragility of plastic crystals was related to the reduced density of minima in the potential energy. However, it is worthwhile to recall that there are some controversy concerning assignments of the main structural process observed in this compound. Consequently, previously derived conclusions might be biased.

Very recently Michl *et al*. have proposed a new, alternative explanation of low fragility of plastic crystals^11^. They clearly demonstrated that the temperature dependence of the number of correlated molecules (*N*_*c*_) is much weaker in such materials with respect to the ordinary glasses. What is more, it was also shown that even for the large *N*_*c*_, the energy barrier for the reorientational motions is pretty low in cyclooctanols, the prototypical ODICs. They rationalized that the lattice strain, which underlined molecular correlations, decreases the effective barrier for the reorientation of the neighboring molecules. However, it should be stressed that authors compared materials differing in chemical structures and intermolecular interactions. That seems to be an important aspect preventing formulation of more general conclusion about the behavior of plastic crystals and ordinary glass formers.

Herein, we report thorough the structural and molecular dynamics studies on 1,6-anhydro-β-D-glucose (levoglucosan), a second compound capable to form ODIC, orientational glass, supercooled liquid and ordinary glass at the same thermodynamic conditions. Herein, it should be mentioned that the molecular dynamics and calorimetric studies on this compound have been reported previously by some of us^12^, Rocha *et al*.^13^ and Tombari *et al*.^14^,^15^. However, the possibility of creation of various degree of disorder by this carbohydrate has not been discussed in any of these articles. Furthermore, the direct comparison of the data obtained for ethanol and levoglucosan provided us an unique opportunity of better understanding a fundamental issue related to the molecular interpretation of the difference between molecular dynamics of materials characterized by various degree of disorder.

X-ray diffraction measurements were carried out for various forms of levoglucosan, namely polycrystalline, plastic crystal, orientational glass, liquids and glass using a laboratory X-ray Rigaku-Denki D/MAX RAPID II-R powder diffractometer attached with a rotating silver anode, an incident beam graphite monochromator and an image plate as two dimensional detector. The experimental set up and the data processing are described in details in refs 16,17. The temperature was controlled using the Oxford Cryostream Plus and Compact Cooler. The dielectric (BDS) measurements were carried out at various temperatures (*T* = 163 K–298 K), in a frequency range from 10^−2^ to 10^6^ Hz, using an Alpha spectrometer (Novocontrol). The sample of 99% purity was obtained from Aldrich.

As discussed above, Rocha *et al*.^13^ and Tombari *et al*.^14^ postulated that 1,6-anhydro-β-D-glucose is a ninth molecule that forms ODIC phase. Interestingly, this state can be created very easily at *T* = 383 K upon heating of the crystal or cooling of the liquid. However, it was also concluded that it is not possible to obtain structural glass in this carbohydrate. We decided to continue their research and found that after application of very fast cooling (molten sample has been directly immersed in liquid nitrogen) levoglucosan supercools and forms a structural glass. Therefore, it is just a second compound that can be prepared as a crystal, plastic crystal, orientational glass, supercooled liquid and ordinary glass.

In Fig. 1, the diffraction data for all investigated phases of levoglucosan measured at indicated temperatures are shown. It should be mentioned that obtained diffractograms enabled us to identify orthorhombic structure of crystal of commercial levoglucosan. In addition, their pair distribution functions are presented in Fig. 2. According to the literature, the diffraction data for disordered materials are usually presented as the structure factor 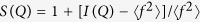, where the scattering vector 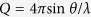, 2*θ* is the scattering angle, *λ* is the wavelength, 
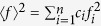
, 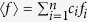, *n* indicates the number of atomic species in the investigated sample, *c*_*i*_ is the concentration and *f*_*i*_ is the atomic scattering factor of the *i*th element, respectively. The diffraction data were then converted to a real space representation in the form of the pair distribution function 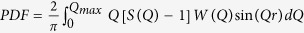, where 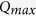 indicates the maximum value of *Q*, achieved in the experiment (here 20 Å^−1^) and 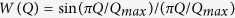 is used in computation in order to minimize truncation oscillations.

As shown in Fig. 1, the structure factors of the plastic crystal, orientational glass, liquid and glass samples exhibit almost the same behavior in the *Q* range from 4 to 20 Å^−1^. For the levoglucosan crystal, sharp diffraction peaks are superimposed on the broad features seen for the other forms of saccharide. The long-period oscillations seen at high *Q*-values can be related to intra-molecular correlations in the atomic arrangement leading to very similar features of the pair distribution functions, *PDF*s, in the range of 0–4.5 Å. These results show that the intra-molecular structure of the levoglucosan molecule remains practically unchanged in the crystal, plastic crystal, orientational glass, liquid and glass states. It is important to note that the *PDF*s for the plastic crystal and orientational glass phases exhibit oscillatory behavior, which extends to about 100 Å indicating longer range ordering of these forms of 1,6-anhydro-β-D-glucose (see right parts of Fig. 2b and c). On the other hand, the oscillations are completely attenuated for the liquid and glass phases at 30 Å and 40 Å, respectively, as it is shown in Fig. 2d and e. The Fourier transforms of the structure factors, computed from the diffraction data limited to the *Q*-range from 1.0 to 1.5 Å, are superimposed on the *PDF*s obtained from the data in the *Q*-range 0–20 Å, as it is presented in Fig. 2b–e. A very good agreement between both curves indicates that in the case of investigated materials the first diffraction peaks are in a direct relationship with longer range inter-molecular correlations. Additional information about a spatial extent of correlations or coherence length can be obtained from the position of the first diffraction peak *Q*_1_ and its width Δ*Q*_1_. The quantity 2π/Δ*Q*_1_ allows estimation of the coherence length for the plastic crystal and orientational glass to be 95 Å on the average of three peaks, that is in agreement with the plots of the *PDF*s displayed in Fig. 2b and c. As it can be clearly seen, the beat-like behavior of the *PDF*s shown in Fig. 2b and c is due to summation of three components with very close frequencies. The coherence lengths obtained for the liquid and glass phases are 30 Å and 39 Å, respectively being also in agreement with decay of the *PDFs* as plotted in Fig. 2d and c. In this context, one should refer to the structural studies reported for ethanol. In this compound, coherence lengths obtained for supercooled and plastic phases are around 10 A and 25 A, indicating that the range of inter-molecular correlations in ethanol and 1,6-anhydro-β-D-glucose is completely different. Another important difference between ethanol and levoglucosan emerged from structural studies. Previously, it was shown that there is noticeable decrease in orientational correlations during passing from the disordered crystal to the glass and liquid in ethanol^18^. On the other hand, such pattern of behavior is not observed for levoglucosan.

Furthermore, the molecular dynamics of 1,6-anhydro-β-D-glucose was investigated by means of BDS. In Fig. 3 dielectric loss spectra obtained in the ODIC and supercooled phases of levoglucosan are presented. In both cases, one dominant structural process, governing glass transition phenomenon, shifting towards lower frequencies with decreasing temperature is observed. In the inset to Fig. 3a, we compared the shape of the structural process measured at the same temperature for carbohydrate prepared in the supercooled liquid (SL) and plastic crystal (PC) phases. The α-loss peak of SL sample was shifted horizontally and vertically to superpose at the maximum with the one obtained for PC. As it can be seen, the distribution of the relaxation times is not the same for both samples. It is slightly broader at the high frequency side for the supercooled 1,6-anhydro-β-D-glucose suggesting most likely more heterogeneous dynamics in this system. In this context, one can recall studies on ethanol where the shape of the most dominant process remained unchanged in ODIC and supercooled states. However, it should be stressed that over the years a lot of controversy arose concerning molecular interpretation of this process. Just to mention that the most intense process detected in this alcohol, might be related to the dynamics of H bonds rather than structural process^19^.

To gain deeper insight into dynamics of PC and SL phases, the measured loss spectra were fitted to Havriliak-Negami (HN) function^20^. In upper panels of Fig. 3 the representative HN fits (solid lines) to the loss spectra measured at both states of levoglucosan are shown. Next, using well known relationship between τ_max_ and HN parameters^21^ the former ones have been determined and plotted as a function of inverse temperature in panel (c) to Fig. 3. Dependencies τ_α_(T) were described by the Vogel-Fulcher-Tammann (VFT equation^22^,^23^,^24^. In addition, relaxation times determined for ethanol are also shown in the inset to Fig. 3(c). As observed, there is a remarkable difference in dynamics of supercooled and plastic phases in alcohol and carbohydrate. Just to mention that in the latter compound just slight difference in the structural dynamics of both considered phases, that vanishes upon approaching the glass transition temperature (*T*_g_ ≈ 245 K), can be detected. Just to mention that herein Tg was defined as temperature at which τ_α_ = 100 s. On the other hand, the dynamics of dominant relaxation process in ethanol is much different in ODIC and supercooled phases. Although similarly as in the case of 1,6-anhydro-β-D-glucose, the transition to the glass and orientational glass occurs at the same temperature *T*_g_ = 97 K^19^. In this context, studies on binary mixtures of succinonitrile with glutaronitrile should be reminded^25^. In this complex system, the glass transition temperature of SL and PC also occurs at the same temperature emphasizing universality of observation of independency of *T*_g_ to the degree of disorder in given material.

Different temperature dependences of the structural relaxation times in ODIC and SL states indicate the difference in the fragility, *m*, of examined carbohydrate as well as ethanol prepared in both forms. In fact, using the following definition of fragility:


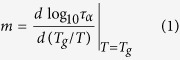


we obtained *m* to be equal to 92, 102 and 40, 60^19^ for Orientationally Disordered Crystalline and supercooled phases of levoglucosan and alcohol, respectively. Recently, Michl *et al*.^11^ claimed that lower fragility of the plastic crystals can be due to lower number of dynamically correlated molecules as well as to the weaker temperature variation in *N*_*c*_. To verify this experimental finding and seek for a closer relation between long range ordering (revealed from structural investigations), dynamical heterogeneities and molecular dynamics, we decided to utilize two methods proposed by Berthier *et al*.^26^,^27^,^28^ and Donth *et al*.^29^,^30^. Briefly, according to the former approach, the approximation of four point susceptibility *χ*_4_(*t*) related to the four point correlation function *G*_4_(*r, t*) can be done with the use of derivative of the two point correlation function. Once KWW function [exp(−(*t/τ*_*α*_)*β*_KWW_)] is applied to describe Φ(*t*), the number of molecules involved in correlated motions *N*_*c*_ can be calculated directly from the formula given by Capaccioli *et al*.^31^:


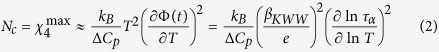


where: Δ*C*_*p*_ is a difference between heat capacity of the supercooled liquid or plastic crystal phase and glass or orientational glass at given *T, k*_*B*_ is a Boltzmann constant and *β*_KWW_ is a stretched parameter. It should be added that Δ*C*_*p*_ (=0.696 J/gK and 0.688 J/gK for SL and PC phase of 1,6-anhydro-β-D-glucose, respectively) were estimated from Differential Scanning Calorimetry measurements (see inset to panel (b) of Fig. 3). Moreover, the last term in equation 2 was calculated from VFT fits (red solid lines) to temperature evolution of structural relaxation times.

The temperature dependences of the number of dynamically correlated molecules are presented in Fig. 3d. As shown, *N*_c_ evolves with temperature in a similar way in both phases reaching value 105 ± 12 and 102 ± 12 at the glass transition for the supercooled and ODIC levoglucosan respectively. Hence, the observed difference seems to be within the experimental uncertainty. It should be mentioned that very similar *N*_c_ (88 ± 9 and 96 ± 9) were determined for leveglucosan prepared in both phases from Donth approach.

Additionally, we also calculated the size of dynamical heterogeneities, *ξ*, which provides information about the length scale of dynamically correlated molecules characterized by the same dynamics or relaxation time. For that purpose following equation that links four point dynamic susceptibility *χ*_*4*_(t) (equation 2) with correlation volume has been used^26^:


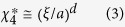


where: *a* (diameter) is equal to 5 Å and *d* defines the shape of the dynamically correlated regions. One can mention that in literature cooperatively rearranging regions of different compact or fractal geometries are considered^32^,^33^,^34^,^35^. In the majority of investigations, *d* varies in the range 2–4. Although, *d* = 3, which reflects cubic or spherical geometry of the dynamically correlated regions is generally taken into account. Due to this reason we assume *d *= 3. In the inset to Fig. 3d, the temperature evolution of *ξ* is presented. It is well seen that *ξ* ≈ 2 nm at the glass transition temperature for levoglucosan prepared in both SL and PC phases As a complementary, this parameter was also evaluated for ethanol *ξ* = 1.8 nm at *T*_g_ using *N*_*c*_ = 195 molecules at *T*_*g*_^26^ and *a *= 3 Å. Our results indicated unquestionably that in contrast to the conclusion derived by Michl *et al*.^11^ *N*_c_ as well as *ξ* evolve in the same manner in PC and SL phases at the same compound. Moreover, taking into account structural and dynamical studies carried out for 1,6-anhydro-β-D-glucose and ethanol, we have an unique opportunity to understand the real molecular mechanism that underlies difference in dynamics of plastic and ordinary glasses appeared. As demonstrated above, the static long range correlations between molecules oscillates around 3–4 nm and 9 nm for supercooled and ODIC states of 1,6-anhydro-β-D-glucose, respectively. What is more, the molecular arrangement in both studied phases is fairly the same. Hence, by simple comparison of length scales of static long range order and dynamical heterogeneities in PC and SL carbohydrate, one can find that the latter quantity is few times lower than the former in both phases. Hence, the length scale of static long range ordering, exceeding significantly *ξ*, seems to be a major factor controlling molecular dynamics of supercooled and plastic phase in levoglucosan. Completely different situation is noted for ethanol. In this alcohol, the length scale of dynamical heterogeneity is almost twice larger than the static intermolecular long range correlations between molecules. Moreover, the significant decrease in orientational correlations was reported when passing from plastic crystal to supercooled state in this material. Therefore, it can be concluded that not heterogeneity alone but rather interplay between length scales of static long range ordering and dynamical heterogeneities as well as internal molecular arrangements and correlations are the most important factors influencing molecular dynamics of supercooled and orientationally disordered crystal states.

In this letter, the molecular dynamics and structural studies were performed on 1,6-anhydro-β-D-glucose, an unique material that form both supercooled liquid and plastic phases at the same thermodynamic conditions. Our combined investigations indicated that the interplay between length scales of static long range inter-molecular correlations and dynamical heterogeneities as well as internal molecular arrangements determines degree of deviation of molecular dynamics of PC and SL phases. This finding is crucial for better understanding of the relationship between varying disorder, static range correlations and molecular dynamics in various materials in the close vicinity of the glass transition temperature.

## Additional Information

**How to cite this article**: Madejczyk, O. *et al*. Interplay between the static ordering and dynamical heterogeneities determining the dynamics of rotation and ordinary liquid phases in 1,6-anhydro-β-D-glucose. *Sci. Rep.* **7**, 42103; doi: 10.1038/srep42103 (2017).

**Publisher's note:** Springer Nature remains neutral with regard to jurisdictional claims in published maps and institutional affiliations.

## Figures and Tables

**Figure 1:**
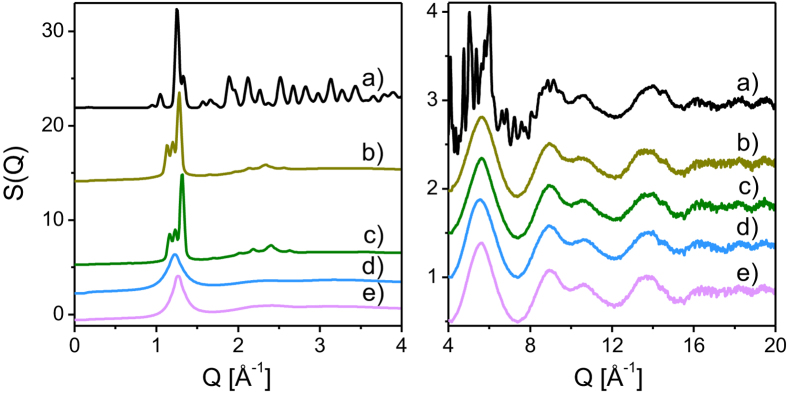
The comparison of the structure factors *S*(Q) obtained from measured diffraction data for the stable levoglucosan crystal at 295 K (a), the plastic phase at 450 K (b), the orientational glass at 230 K (c), the liquid at 470 K (d), and the glass at 230 K (e).

**Figure 2:**
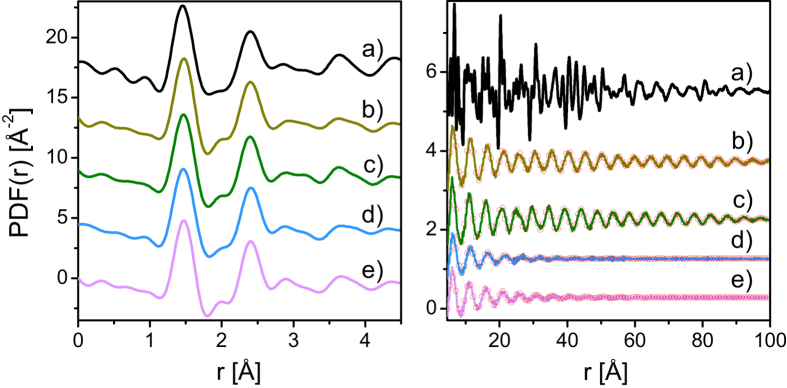
The comparison of the pair distribution functions, *PDF*(*r*), computed from the data displayed in Fig. 1 for the stable levoglucosan crystal at 295 K (a), the plastic phase at 450 K (b), the orientational glass at 230 K (c), the liquid at 470 K (d), and the glass at 230 K (e). The open circles on the right part represent the Fourier transforms of the structure factors computed for the *Q*-range from 1.0 Å to 1.5 Å.

**Figure 3:**
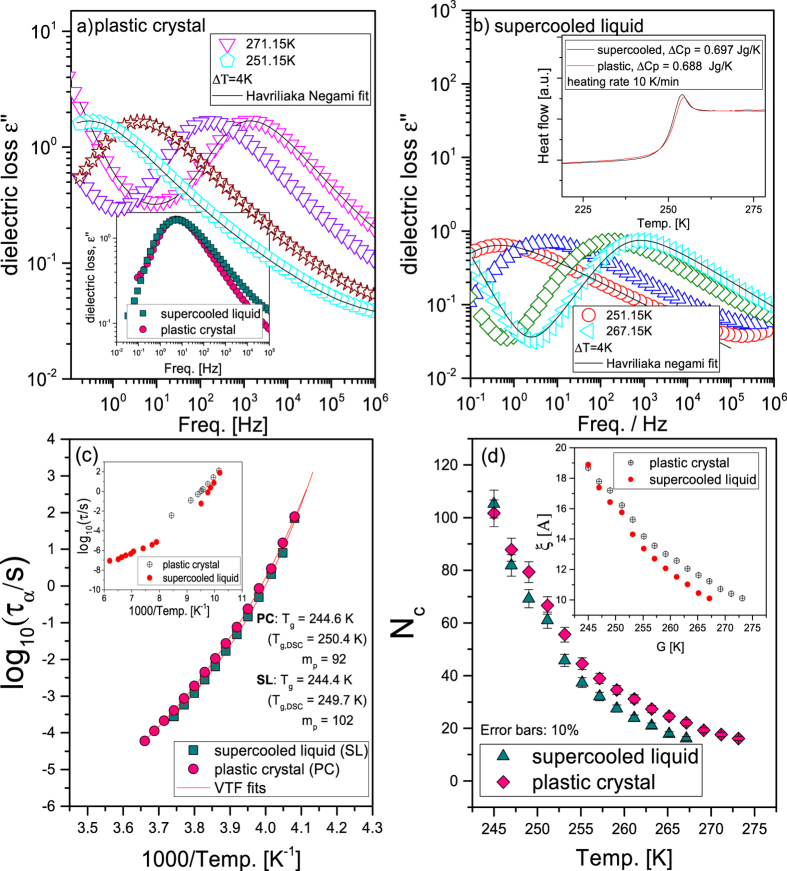
Dielectric loss spectra measured in plastic crystal (**a**) and supercooled phase (**b**); Solid lines represent HN fits. In the inset to panel (a) a comparison of the shape of the structural process measured in both phases and is shown. In the inset to panel (b) heat capacity jump at the glass transition temperature obtained from DSC measurements for SL and PC forms of saccharide. The temperature dependence of the structural relaxation times (**c**), where solid lines are the best VFT fits; The inset presents relaxation times obtained by Benkhof *et al.^19^* for ethanol in different phases. The number of dynamically correlated molecules is plotted vs temperature in panel (d), while the inset demonstrates the temperature evolution of length scale of dynamic heterogeneity *ξ* in both phases of levoglucosan.
